# Improvements and new functionalities of UNRES server for coarse-grained modeling of protein structure, dynamics, and interactions

**DOI:** 10.3389/fmolb.2022.1071428

**Published:** 2022-12-14

**Authors:** Rafał Ślusarz, Emilia A. Lubecka, Cezary Czaplewski, Adam Liwo

**Affiliations:** ^1^ Faculty of Chemistry, University of Gdańsk, Fahrenheit Union of Universities in Gdańsk, Gdańsk, Poland; ^2^ Faculty of Electronics, Telecommunication and Informatics, Gdańsk University of Technology, Fahrenheit Union of Universities in Gdańsk, Gdańsk, Poland

**Keywords:** protein-structure modeling, coarse graining, UNRES model of polypeptide chains, molecular dynamics, data-assisted simulations

## Abstract

In this paper we report the improvements and extensions of the UNRES server (https://unres-server.chem.ug.edu.pl) for physics-based simulations with the coarse-grained UNRES model of polypeptide chains. The improvements include the replacement of the old code with the recently optimized one and adding the recent scale-consistent variant of the UNRES force field, which performs better in the modeling of proteins with the *β* and the *α*+*β* structures. The scope of applications of the package was extended to data-assisted simulations with restraints from nuclear magnetic resonance (NMR) and chemical crosslink mass-spectroscopy (XL-MS) measurements. NMR restraints can be input in the NMR Exchange Format (NEF), which has become a standard. Ambiguous NMR restraints are handled without expert intervention owing to a specially designed penalty function. The server can be used to run smaller jobs directly or to prepare input data to run larger production jobs by using standalone installations of UNRES.

## 1 Introduction

Coarse-grained simulations are now a well established methodology with which to study large systems at large time scales. Compared to all-atom simulations, they offer a 1,000-fold or greater extension of simulation time scale (Khalili et al., 2005a; Kmiecik et al., 2016), enabling us to simulate biologically important events in real time (Kmiecik et al., 2016; Liwo et al., 2020). This gain, however, is achieved at the expense of accuracy compared to using the all-atom representation. One reason for decreasing the accuracy is a lower resolution of coarse-grained models compared to that of all-atom models. However, a more important reason is that most of the coarse-grained force fields are constructed by analogy to the all-atom ones, which is not correct from the physics point of view. The physical origin of a coarse-grained force field is the potential of mean force of a given system, in which the degrees of freedom not explicitly considered in the model are averaged out (Liwo et al., 2001; Ayton et al., 2007). Consequently, the interaction potentials have a lower symmetry than the spherical symmetry and taking into account multibody terms is a necessity, as opposed to all-atom force fields (Liwo et al., 2001; Liwo et al., 2020). Moreover, many coarse-grained force fields are derived from structural data rather than in a bottom-up manner from all-atom energy surfaces (Kmiecik et al., 2016; Liwo et al., 2020), which makes the derived energy terms more difficult to understand and control. On the other hand, including even sparse restraints from experiments such as, e.g., nuclear magnetic resonance (NMR), chemical cross link mass spectroscopy (XL-MS), and small angle X-ray diffraction data (SAXS) can reduce the impact of force-field inaccuracy on the results. Therefore, data-assisted simulations are often carried out (Bonomi et al., 2016; Brodie et al., 2017; Karczyńska et al., 2018; Fajardo et al., 2019; Bottaro et al., 2020; Czaplewski et al., 2021).

For proteins, a number of coarse-grained models are available, namely AWSEM (Davtyan et al., 2012), OpenAWSEM (Lu et al., 2021), CABS (Kolinski, 2004), MARTINI (Marrink and Tieleman, 2013; Marrink et al., 2022), SIRAH (Darré et al., 2015), and UNRES which, under the name UNICORN was upgraded to treat proteins, nucleic acids, and polysaccharides (Liwo et al., 2014; Sieradzan et al., 2022a). The MARTINI model is the most general one and the package enables automatic coarse-graining of any system with no or little user intervention. On the other hand, only AWSEM, CABS, and UNRES are capable of folding proteins. For peptides and proteins the OPEP model (Chebaro et al., 2012) is also used, which has the folding capacity. For protein-structure prediction, the ROSETTA model (Rohl et al., 2004) has been used with great success. However, this model makes extensive use of bioinformatics-based filters and is designed to locate the candidate predictions as structure with lowest potential energies. The above models differ in the degree of coarse graining, type of potential and methods of conformational search. AWSEM, ROSETTA, and OPEP use all-atom backbone, while the backbone is coarse-grained in CABS, MARTINI, SIRAH, and UNRES. CABS is entirely based on statistical potentials, while the other models mentioned here have physics-based components.

The structure-based coarse-grained models constitute another class of models, in which the native structure is the global minimum. There are two types of these models, namely the Gō-like and elastic-network models. In the Gō-like models (Taketomi et al., 1975; Hills and Brooks, 2009), long-range residue-residue contacts are assigned a potential with energy minimum, while non-native contacts are assigned all-repulsive potentials. In the elastic-network models harmonic or anharmonic potentials (sometimes double-well potentials) are imposed on all residue pairs (Sinitskiy and Voth, 2008; Trylska, 2010; Kmiecik et al., 2018).

Compared to established all-atom packages, the installation of the respective software, preparing the input data, running calculations, and processing the results is more difficult. To facilitate job data preparation and running simulations, web servers were created for the AWSEM (Jin et al., 2020), CABS (CABS-fold) (Blaszczyk et al., 2013), OPEP (PEP-FOLD3) (Lamiable et al., 2016) and UNRES/UNICORN (UNRES web server) (Czaplewski et al., 2018) models. Protein structure prediction with the ROSETTA model can be accomplished by using the ROBETTA web server (Kim et al., 2004). However, these servers do not run data-assisted simulations except for the UNRES server.

The UNRES model of polypeptide chains (Liwo et al., 2014; Sieradzan et al., 2022a) developed in our laboratory is a heavily coarse-grained model, with only two interaction sites per amino-acid residue, namely a united peptide group and a united side chain. The effective energy function has been developed on the physical basis, by expressing the potential of mean force of a system in terms of Kubo cluster cumulant functions (Kubo, 1962), which are approximated analytically (Liwo et al., 2001; Sieradzan et al., 2017) by Kubo cluster cumulants. Owing to this method of derivation, the respective interaction potentials are dependent on both site distance and site orientation and the expressions for multibody terms, which are essential to reproduce regular secondary structures (Kolinski and Skolnick, 1992; Liwo et al., 2001), have been derived. UNRES has been successful in protein-structure prediction, in studying protein-folding dynamics and thermodynamics, and in solving biological problems (Sieradzan et al., 2022a).

The UNRES package is accessible as a standalone version (https://unres.pl) and as a web server, with which small production jobs can be run. The first version (Czaplewski et al., 2018) was released 4 years ago. That version ran a then state-of-the art variant of the UNRES force field, which had a significant predictive power but produced too compressed *β*-strands (Krupa et al., 2017a). The first version of the server could also handle SAXS restraints. Recently, we developed a scale-consistent theory of coarse-grained force-field derivation (Sieradzan et al., 2017; Liwo et al., 2020) and a new version of UNRES (Liwo et al., 2019), which handles the *β*-strand and loop geometry much better, producing structures with a higher resolution, as demonstrated in the CASP13 (Lubecka et al., 2019) and CASP14 (Antoniak et al., 2021) community-wide experiments of the assessment of methods for protein-structure prediction (https://predictioncenter.org). The UNRES package has also been enhanced with the XL-MS (Fajardo et al., 2019; Kogut et al., 2021) and NMR (Lubecka and Liwo, 2021, 2022) data-assisted-calculation capacities, which have recently been included in the server. Finally, the UNRES code has recently been heavily optimized for memory and speed (Sieradzan et al., 2022b) and the optimized code has been included in the server. The new features of the UNRES server are described in this article. Examples are provided to illustrate the new data-assisted-calculation features.

## 2 Materials and methods

### 2.1 UNRES model and force field

**FIGURE 1:**
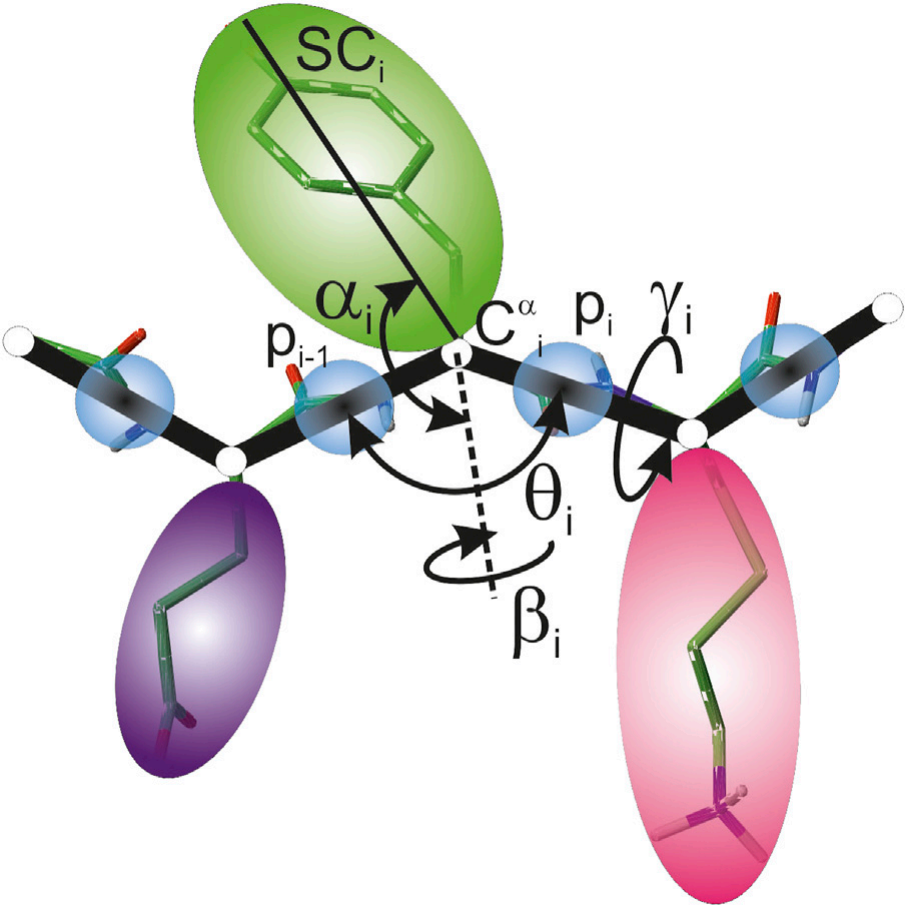
UNRES model of polypeptide chains. The interaction sites are united peptide groups located between the consecutive *α*-carbon atoms (light-blue spheres) and united side chains attached to the *α*-carbon atoms (spheroids with different colors and dimensions). The backbone geometry of the simplified polypeptide chain is defined by the C^*α*^⋯ C^*α*^⋯ C^*α*^ virtual-bond angles *θ* (*θ*_*i*_ has the vertex at $C_{i}^{\alpha }$ ) and the C^*α*^⋯ C^*α*^⋯ C^*α*^⋯ C^*α*^ virtual-bond-dihedral angles *γ* (*γ*_*i*_ has the axis passing trough $C_{i}^{\alpha }$ and $C_{i+1}^{\alpha }$ ). The local geometry of the *i*th side-chain center is defined by the polar angle *α*_*i*_ (the angle between the bisector of the respective angle *θ*_*i*_ and the $C_{i}^{\alpha }\cdots \;$ SC_*i*_ vector) and the azimuth angle *β*_*i*_ (the angle of counter-clockwise rotation of the $C_{i}^{\alpha }\cdots \;$ SC_*i*_ vector about the bisector from the $C_{i-1}^{\alpha }\cdots \;$ $C_{i}^{\alpha }\cdots \;$ $C_{i+1}^{\alpha }$ plane, starting from $C_{i-1}^{\alpha }$ ). For illustration, the bonds of the all-atom chains, except for those to the hydrogen atoms connected with the carbon atoms, are superposed on the coarse-grained picture. Reproduced with permission from Zaborowski et al., 2015, J. Chem. Inf. Model., 55, 2050 (2015). Copyright 2015 American Chemical Society.

In the UNRES model, a polypeptide chain is represented by a sequence of *α*-carbon (C^*α*^) atoms linked with virtual bonds, with peptide groups (p) located halfway between the consecutive C^*α*^s and united side chains (SC) attached to the C^*α*^s with the C^*α*^⋯ SC virtual bonds (Figure 1). Only the united peptide groups and the united side chains are interaction sites, while the C^*α*^s assist in chain-geometry definition.

The UNRES energy function is expressed by Eq. 1.

$$\begin{array}{ccc} U=w_{SC}\underset{i<j}{\sum }U_{SC_{i}SC_{j}}+w_{\mathrm{SCp}}\underset{i\neq j}{\sum }U_{SC_{i}p_{j}}+w_{pp}^{\mathrm{V DW}}\underset{i<j-1}{\sum }U_{p_{i}p_{j}}^{\mathrm{V DW}}+w_{pp}^{el}f_{2}\left(T\right)\underset{i<j-1}{\sum }U_{p_{i}p_{j}}^{el}\\ & + & w_{\mathrm{tor}}f_{2}\left(T\right)\underset{i}{\sum }U_{\mathrm{tor}}\left(\gamma_{i},\theta_{i},\theta_{i+1}\right)+w_{b}\underset{i}{\sum }U_{b}\left(\theta_{i}\right)+w_{\mathrm{rot}}\underset{i}{\sum }U_{\mathrm{rot}}\left(\theta_{i},{\hat{r}}_{SC_{i}}\right)\\ & + & w_{\mathrm{bond}}\underset{i}{\sum }U_{\mathrm{bond}}\left(d_{i}\right)+w_{ssbond}\underset{i}{\sum }U_{\mathrm{ssbond}}\left(d_{i}^{SS}\right)\\ & + & w_{\mathrm{corr}}^{\left(3\right)}f_{3}\left(T\right)\underset{i<j-1}{\sum }U_{\mathrm{corr;ij}}^{\left(3\right)}+w_{\mathrm{turn}}^{\left(3\right)}f_{3}\left(T\right)\underset{i}{\sum }U_{\mathrm{turn;i}}^{\left(3\right)} \end{array}$$ (1)

where the terms $U_{SC_{i}SC_{j}}$ are sidechain-sidechain interaction energies represented by the modified Gay-Berne potentials (Liwo et al., 1997), $U_{SC_{i}p_{j}}$ are excluded-volume potentials that prevent the collapse of the united side chains on the backbone, $U_{p_{i}p_{j}}^{\mathrm{V DW}}$ (with spherical symmetry) and $U_{p_{i}p_{j}}^{el}$ (with axial symmetry) are the non-bonded and mean-field-electrostatic interaction potentials of united peptide groups, *U*_*bond*_, are the bond-deformation potentials, *U*_*b*_ and *U*_*tor*_ are the backbone-virtual-bond-angle and the backbone-virtual-bond-torsional potentials, respectively, *θ*_*i*_ and *γ*_*i*_ denoting the virtual-bond- and virtual-bond-dihedral angles, respectively (Figure 1), *U*_*rot*_ are the side-chain-rotamer potentials, in which ${\hat{r}}_{SC_{i}}$ denotes the local coordinates of the unit vector pointing from $C_{i}^{\alpha }$ to SC_*i*_, while $U_{\mathrm{corr}}^{\left(3\right)}$ and $U_{\mathrm{turn}}^{\left(3\right)}$ are multibody terms that account for the coupling of the backbone-local and backbone-electrostatic interactions (Liwo et al., 2001; Sieradzan et al., 2017). *U*_*ssbond*_ denotes the terms that account for the energetics of disulfide bonds, including their formation and breaking (Chinchio et al., 2007; Krupa et al., 2017b). The solvent is implicit in the interaction potentials and the present parameterization corresponds to physiological pH. The *w*s are the weights of the energy terms and have been determined, along with some other parameters, by maximum-likelihood calibration of the force field (Liwo et al., 2019).

The factors *f*_*n*_(*T*) account for the dependence of the force-field terms that correspond to higher-order terms in the Kubo cluster-cumulant expansion on temperature (Liwo et al., 2007), as given by Eq. 2.

$$f_{n}\left(T\right)=\frac{\ln\left[\exp\left(1\right)+\exp\left(-1\right)\right]}{\ln\left\{\exp\left[{\left(T/T_{○}\right)}^{n-1}\right]+\exp\left[-{\left(T/T_{○}\right)}^{n-1}\right]\right\}}$$ (2)

where *T*_◦_ = 300 K.

As mentioned in the *Introduction*, the UNRES effective energy function originates from the potential of mean force of polypeptide chains in water, which is represented (Liwo et al., 2001; Sieradzan et al., 2017) by a truncated series of Kubo cluster cumulant functions (Kubo, 1962). Consequently, it has the sense of free energy and depends on temperature (Liwo et al., 2007). The energy terms in the present version of UNRES have been derived based on our recently developed scale-consistent theory of coarse graining (Sieradzan et al., 2017), in which the atomistic details are rigorously embedded in the effective interactions potentials. As a result of the application of this theory, the torsional potentials depend not only on the virtual-bond-dihedral angles but also on the adjacent virtual-bond angles, tending to zero when a chain fragment becomes linear. This feature of the torsional potentials eliminates the problem of their indefiniteness in such situations (Sieradzan et al., 2017). Similarly, the terms accounting for the correlation of the backbone-electrostatic and backbone-local interactions, which formerly depended on the distance and orientation of the peptide groups involved and the backbone virtual-bond-dihedral angles located at them, now also depend on the virtual-bond angles. These features greatly improved the accuracy of the modeling of *β* and *α*+*β* proteins (Liwo et al., 2019). The latest NEWCT-9P variant of the UNRES force field implemented in the upgraded UNRES server has been parameterized by a maximum likelihood method (Zaborowski et al., 2015) with nine proteins of all secondary-structure types (Liwo et al., 2019).

The rigorous physics-based derivation of the effective energy terms outlined above distinguishes UNRES from other coarse-grained force fields, in which the energy terms have been constructed by analogy to all-atom energy terms or on a heuristic basis (Kmiecik et al., 2016; Liwo et al., 2020). Owing to this derivation, the UNRES energy terms can be tracked down to elemental atomic interactions. The rigorous derivation also enabled us to capture the dependence of the effective potentials on site orientation and to derive the expressions for correlation terms. Details of the UNRES model and force field are available in the references cited (Liwo et al., 2014; Sieradzan et al., 2017; Liwo et al., 2019; Sieradzan et al., 2022a).

### 2.2 Molecular dynamics and its extensions with UNRES

The engine of the conformational search with UNRES is molecular dynamics, which has been implemented using the Lagrange formalism (Khalili et al., 2005b,a). Due to the axial symmetry of the interacting sites, the equations of motion are more complicated than those of the coarse-grained models that use spherical potentials (AWSEM, MARTINI, and SIRAH). The inertia matrix is not diagonal; however, it is a constant matrix. In our recent work (Sieradzan et al., 2022b), we reduced the inertia matrix to a five-band form, this saving both memory and computing time. MD simulations with UNRES can be run in the microcanonical (NVE) and canonical (NVT) mode, the latter with the Berendsen or the Langevin thermostat. For better conformational search, replica-exchange (REMD) (Hansmann, 1997) and multiplexed replica exchange molecular dynamics (Rhee and Pande, 2003) have been implemented in UNRES (Czaplewski et al., 2009). A binless version of the weighted histogram analysis method (WHAM) (Kumar et al., 1992) has been implemented (Liwo et al., 2007) to process the results of REMD/MREMD simulations in order to calculate ensemble-averaged properties and to determine conformational ensembles at the desired temperatures.

The UNRES molecular-dynamics code has been parallelized (Liwo et al., 2010) and its parallel implementation has been heavily upgraded and optimized recently (Sieradzan et al., 2022b). With the new code, molecular dynamics simulations of protein systems with sizes exceeding 100,000 amino-acid residues, reaching over 1 ns/day with 24 cores are feasible. Compared to the previous version, the new code is 2–50 times faster, depending on protein size and degree of parallelization (Sieradzan et al., 2022b).

### 2.3 Experimental restraints

As mentioned in the Introduction, UNRES can handle the experimental restraints from NMR, XL-MS, and SAXS experiments. These are included by adding the respective penalty functions to the UNRES energy, as given by Eq. 3.

$$V=U_{\mathrm{UNRES}}+w_{\mathrm{NMR}}^{\theta }V_{\mathrm{NMR}}^{\theta }+w_{\mathrm{NMR}}^{\gamma }V_{\mathrm{NMR}}^{\gamma }+w_{\mathrm{NMR}}^{\mathrm{dist}}V_{\mathrm{NMR}}^{\mathrm{dist}}+w_{XL-MX}^{\mathrm{dist}}V_{XL-MS}^{\mathrm{dist}}+w_{\mathrm{SAXS}}V_{\mathrm{SAXS}}$$ (3)

where $V_{\mathrm{NMR}}^{\theta }$ and $V_{\mathrm{NMR}}^{\gamma }$ are the penalty terms corresponding to virtual-bond angles *θ* and virtual-bond-dihedral angles *γ* (cf. Figure 1), respectively, derived from NMR data, $V_{\mathrm{NMR}}^{\mathrm{dist}}$ is the penalty term corresponding to the interproton distances derived from NMR data, $V_{XL-MS}^{\mathrm{dist}}$ is the distance-penalty term corresponding to crosslink restraints, *V*_*SAXS*_ is the SAXS-restraint term, and the *w*s are the weights of the respective penalty terms. The specific terms are described in Sections 2.3.1–2.3.3.

#### 2.3.1 NMR restraints

As follows from Eq. 3, the NMR restraints consist of the angular and the distance terms. Chemical shifts (CS) and coupling-constants data provide the restraints on the backbone dihedral angles *ϕ* and *ψ*. These are converted to the restraints on the *θ* and the *γ* angles (cf. Figure 1) by using the formulas from (Nishikawa et al., 1974). The penalty function is a flat-bottom function as given by Eqs 4–7 (Lubecka and Liwo, 2022).

$$V_{\mathrm{NMR}}^{\theta }=g\left(\theta ,\theta_{l},\theta_{u}\right)$$ (4)

$$V_{\mathrm{NMR}}^{\gamma }=g\left(\gamma ,\gamma_{l},\gamma_{u}\right)$$ (5)

with

$$g\left(x,x_{l},x_{u}\right)=\left\{\begin{array}{cc} \frac{1}{4}{\left(\delta +\frac{x_{u}-x_{l}}{2}\right)}^{4}\; & for\;\delta <\frac{x_{l}-x_{u}}{2}\\ 0\; & for\;\frac{x_{l}-x_{u}}{2}<\delta <\frac{x_{u}-x_{x}}{2}\\ \frac{1}{4}{\left(\delta +\frac{x_{l}-x_{u}}{2}\right)}^{4}\; & for\;\delta >\frac{x_{u}-x_{l}}{2} \end{array}\right.$$ (6)

$$\delta =\left(x-\frac{x_{l}+x_{u}}{2}\right)\mathrm{mod}2\pi$$ (7)

where *θ*_*l*_, *θ*_*u*_, *γ*_*l*_, and *γ*_*u*_ are the lower and upper boundaries on the virtual-bond angles *θ* and virtual-bond-dihedral angles *γ*, respectively (which are calculated from the boundaries on the *ϕ* and *ψ* backbone dihedral angles).

To include the distance restraints, the positions of the protons are estimated first by using our recently developed ESCASA algorithm (Lubecka and Liwo, 2021), which is based on analytical formulas. This algorithm also provides analytical gradients of the estimated proton positions with respect to coarse-grained coordinates. A flat-bottom penalty function with a mild slope is imposed on each interproton distance estimated by NMR, as given by Eq. 8.

$$V_{\mathrm{cont}}\left(d,d_{l},d_{u},A\right)=\left\{\begin{array}{cc} A\frac{{\left(d-d_{l}\right)}^{4}}{\sigma^{4}+{\left(d-d_{l}\right)}^{4}}\left[1+\kappa \ln\cosh\left(d-d_{l}\right)\right]\; & for\;d<d_{l}\\ 0\; & for\;d_{l}\leq d\leq d_{u}\\ A\frac{{\left(d-d_{u}\right)}^{4}}{\sigma^{4}+{\left(d-d_{u}\right)}^{4}}\left[1+\kappa \ln\cosh\left(d-d_{u}\right)\right]\; & for\;d>d_{u} \end{array}\right.$$ (8)

where *d* is a proton-proton distance estimated from an UNRES structure, *d*_*l*_ and *d*_*u*_ are the lower and upper distance boundaries, respectively, which are taken from NMR data, *σ* is the thickness of the transition region between zero and maximum restraint height, *A* is the height of the restraint well, and *κ* is the slope of the restraint at large distances. The default values of *σ* and *A* are 0.5 Å and 1.0 kcal/mol, respectively. The original penalty function from our earlier work (Sieradzan and Jakubowski, 2017; Lubecka and Liwo, 2019) corresponds to *κ* = 0 and quickly approaches the asymptote *A*, contributing virtually no force when *d* ≫ *d*_*u*_. Thus, the penalty terms do not force incompatible restraints (which usually correspond to wrongly predicted contacts), preventing a simulation from producing non-protein-like structures. With a small *κ* > 0 (default 0.01), the right asymptote is *A*+*κ*(*d*−*d*_*u*_), which provides a small gradient at large distances, thus mildly guiding the search towards satisfying the restraint but not forcing it if incompatible with the other restraints.

To treat ambiguous restraints, the restraint-penalties of an ambiguous set are put into a log-exp function, which has the shape of intersecting gorges and, therefore, takes the minimum value regardless of whether one only or more restraints of an ambiguous-restraint sets are satisfied (Eq. 9) (Lubecka and Liwo, 2022).

$$V_{\mathrm{NMR}}^{\mathrm{dist}}\left(\left\{d\right\};d_{l},d_{u},A\right)=-\frac{1}{\alpha }\ln\left\{\overset{n_{\mathrm{amb}}}{\underset{i=1}{\sum }}\exp\left[-\alpha V_{\mathrm{cont}}\left(d_{i};d_{l},d_{u},A\right)\right]\right\}$$ (9)

where {*d*} is the set of distances corresponding to a given ambiguous restraint, *α* is an arbitrary parameter, and *V*_*cont*_(*d*_*i*_; *d*_*l*_, *d*_*u*_, *A*) is defined by Eq. 8. With *α* large enough (default 20), *V*_*NMR*_({*d*}; *d*_*l*_, *d*_*u*_, *A*) is nearly 0, regardless of whether only one or all restraints of the ambiguous set are satisfied. Thus, the restraints of an ambiguous set, which are incompatible with the structure are eliminated.

In this work, we optimized the calculation of the ambiguous penalty function given by Eq. 9 by considering only the exponentials for which the distance-penalty is not too big to make them close to zero and parallelized the evaluation of the penalty function to achieve load balance.

Apart from UNRES, the NMR-data-assisted simulations can be run with the CABS (CABS-NMR) (Latek and Koliński, 2011) and ROSETTA (CS-ROSETTA) (Nerli and Sgourakis, 2019) coarse-grained models. However, ROSETTA uses all-atom backbone, with which the calculation of interproton distances and backbone dihedral angles is straightforward, while the procedure implemented in CABS-NMR requires the conversion from the coarse-grained to the all-atom representation to evaluate the NMR-penalty term Latek and Koliński (2011), which involves additional computational effort and restricts the use of the approach to Monte Carlo simulations. As opposed to this, the ESCASA algorithm expresses the proton coordinates analytically in terms of coarse-grained geometry and, consequently, enables us to compute analytical forces due to the NMR-penalty term which, in turn, makes possible to implement it in molecular dynamics simulations (Lubecka and Liwo, 2021).

#### 2.3.2 XL-MS restraints

Three types of XL-MS restraints have been implemented in UNRES. The restraints of the first type have the form of a flat-bottom bounded function imposed on the respective C^*α*^⋯ C^*α*^ or SC⋯ SC distances (Eq. 8 in which *κ* = 0). These were designed for the non-specific crosslinks proposed by Rappsilber (2011). Because the right boundary of the distance between the crosslinked residues is 25 Å, they do not perform well in data-assisted simulations (Fajardo et al., 2019). The crosslinks of the second type are statistical pseudopotentials (Fajardo et al., 2019; Kogut et al., 2021) that are based on C^*α*^ ⋯ C^*α*^-distance distributions of crosslinked residue pairs from known proteins determined by Leitner et al. (2014) (Eq. 10). The cross linking agents are the adipic-acid (ADH) and the pimelic-acid (PDH) hydrazides linking the acidic side chains and disuccinimidyl suberate (DSS), which links the side chains of lysine residues. Zero-length (ZL) crosslinks that occur between basic and acidic side chains or involve the N-terminal amino groups or the C-terminal carboxyl groups are also included. These restraints perform well in the data-assisted modeling of protein structure with UNRES (Leitner et al., 2014; Kogut et al., 2021).

$$W\left(d\right)=-ART\ln\left\{\left[a+b{\left(\frac{d}{\sigma }\right)}^{4}\right]\exp\left(-\frac{d^{2}}{2\sigma^{2}}\right)+c\right\}$$ (10)

where *d* is the distance between the C^*α*^-atoms of the crosslinked residues, *a*, *b*, *c*, and *σ* are crosslink-specific parameters, *R* is the universal gas constant, *T* is the absolute temperature; we assumed *T* = 298 K, hence *RT* = 0.591 kcal/mol, and *A* (default 15) is the weight of the potential, which is assigned the confidence of the crosslink.

Restraints of the third type are the pseudopotentials imposed on the distances between the ends of the acidic residue side chains linked by ADH or PDH or the ends of the lysine side chains linked by glutaric acid dipentyloamide (BS^2^) or suberic acid dipentyloamide (BS^3^) and the virtual-bond angles and the virtual-bond-dihedral angle of the crosslinked C^*α*^⋯X⋯X⋯C^*α*^ moiety, where *X* means the end of the respective side chain (Kogut et al., 2021), as given by Eqs 11–14. These restraint potentials performed well in data-assisted UNRES simulations (Kogut et al., 2021).

$$\begin{array}{ll} V\left(d_{X_{i}},d_{X_{j}},d_{X_{i}Xj},\theta_{X_{i}},\theta_{X_{j}},\gamma_{X_{i}X_{j}}\right) & =V_{d}\left(d_{X_{i}}\right)+V_{d}\left(d_{X_{j}}\right)+V_{d}\left(d_{X_{i}X_{j}}\right)+V_{\theta }\left(\theta_{X_{i}}\right)+V_{\theta }\left(\theta_{X_{j}}\right)+\\ & V_{\gamma }\left(\gamma_{X_{i}X_{j}},\theta_{X_{i}},\theta_{X_{j}}\right) \end{array}$$ (11)

**FIGURE 2:**
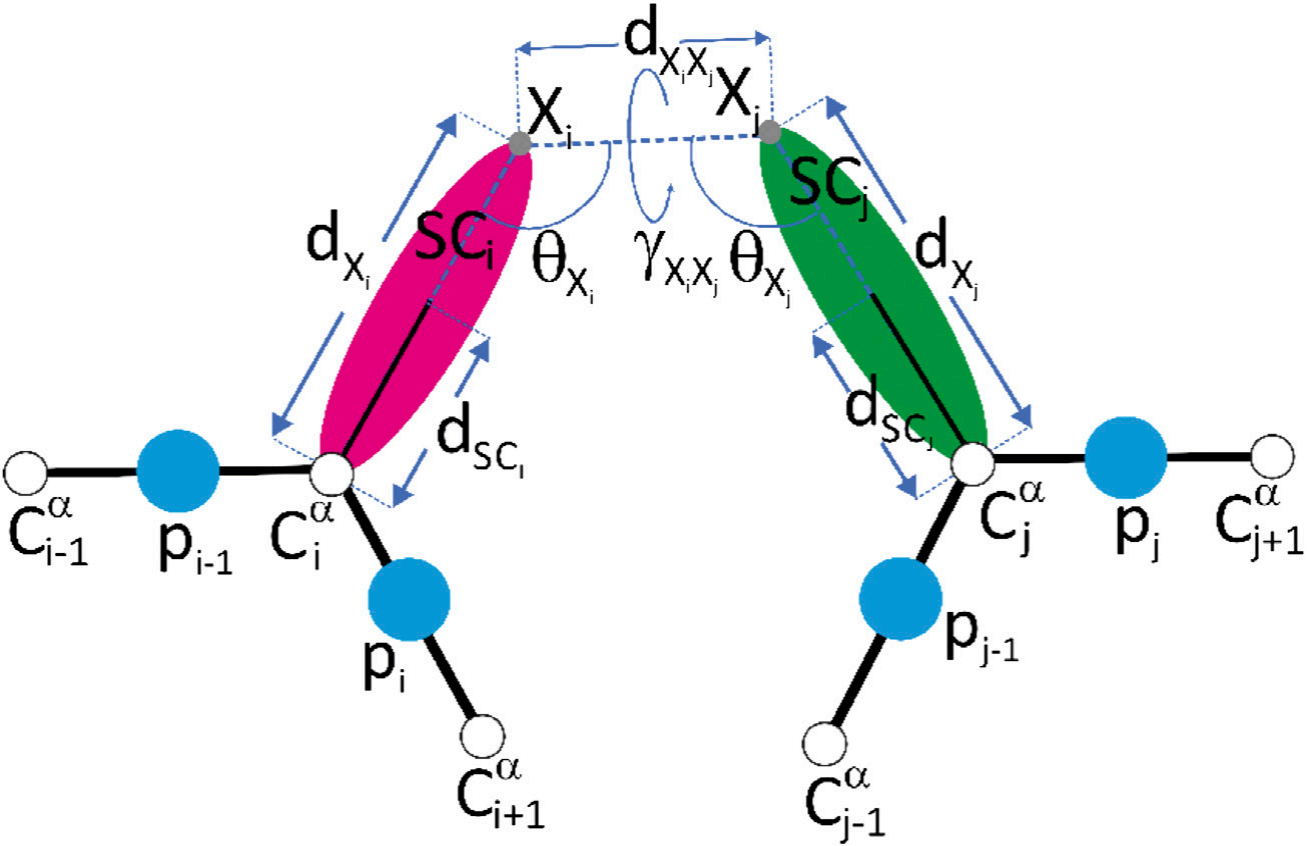
A scheme of the representation of crosslink restraints between residues with indices *i* and *j*, respectively, in the UNRES model. The C^*α*^ atoms are shown as white spheres, the united side chains (SC) are shown as colored spheroids, and the united peptide groups (p) are shown as blue spheres. The crosslinkable side chains (aspartic acid, glutamic acid or lysine) are linked with adipic/pimelic acid (ADH or PDH) dihydrazide or by the glutaric/suberic acid (BS^2^G or BS^3^), respectively. The link is anchored in (approximately) the positions of the side-chain carbonyl-carbon atoms of Asp or Glu (for the ADH or PDH crosslinks) or in the positions of the lysine side-chain nitrogen atoms (for the BS^2^G or BS^3^ crosslinks), respectively. The anchor points (indicated with “X” and light-gray spheres) are located on the C^*α*^⋯ SC axes of the UNRES residues. The geometric parameters on which the respective pseudopotentials depend [Eqs 11–14] are also shown in the Figure. Reproduced with permission from Kogut et al. (2021), J. Comput. Chem. 42, 2054 (2021). Copyright 2021 John Wiley and Sons.

Vdd=∏j=1Ndaj+12kjd−dj°2∑j=1Nd∏j=1j≠iNdaj+12kjd−dj°2 (12)

$$V_{\theta }\left(\theta \right)=a_{○}+\overset{N_{\theta }}{\underset{j=1}{\sum }}a_{j}{\left(\cos\theta \right)}^{j}+b_{j}{\left(\sin\theta \right)}^{j}$$ (13)

$$V_{\gamma }\left(\gamma ,\theta_{1},\theta_{2}\right)=V_{○}+\overset{N_{\gamma }}{\underset{j=1}{\sum }}c_{j}{\left(\sin\theta_{1}\right)}^{j}{\left(\sin\theta_{2}\right)}^{j}\cos\left(j\gamma \right)$$ (14)

where *N*_*d*_, *N*_*θ*_, and *N*_*γ*_ are the numbers of terms in the expressions for the virtual-bond-length, virtual-bond-angle, and virtual-bond-dihedral-angle potentials and the other symbols except for geometric variables ( $d_{X_{i}}$ , $\theta_{X_{i}}$ , $\theta_{X_{j}}$ , and $\gamma_{X_{i}X_{j}}$ ) are adjustable parameters, which have been determined in (Kogut et al., 2021). The geometric variables are illustrated in Figure 2.

A full menu of crosslink-restraint potentials, which include the statistical potentials given by Eq. 10 and the MD-derived potentials given by Eqs 12–14 is available only with UNRES. Implementations with other coarse-grained models such as, e.g., ROSETTA (Nerli and Sgourakis, 2019) and MEDUSA (Brodie et al., 2017) include only the square-well type contact potentials similar to that given by Eq. 8.

#### 2.3.3 SAXS restraints

In our approach (Karczyńska et al., 2018), the SAXS restraint-penalty function is based on the experimental distance distribution, *P* *^exp^* (*r*), determined by the Fourier transform of the intensity, *I*(*q*), where *r* is the distance and *q* = 4*π* sin *θ*/*λ*, *θ* being the scatter angle and *λ* being the wavelength respective restraint function. The penalty function, *V*_*SAXS*_, is a maximum-likelihood function, as given by Eq. 15.

$$V_{\mathrm{SAXS}}=-\overset{d_{max}}{\underset{0}{\int }}P^{\mathrm{SAXS}}\left(r\right)\ln P^{\mathrm{calc}}\left(r\right)dr\approx -\Delta r\overset{M}{\underset{k=1}{\sum }}P^{\mathrm{SAXS}}\left(r_{k}\right)\ln P^{\mathrm{calc}}\left(r_{k}\right)$$ (15)

where *r*_*k*_ is the distance at the center of the *k*th bin of the histogram of the distance distribution from SAXS measurements, *M* is the number of bins, *P* ^*SAXS*^(*r*) is the value of the probability distribution determined by SAXS at *r*, *P* ^*calc*^(*r*) is the value of the probability distribution calculated from simulations at *r*, and *d* *_max_* is the maximum distance in the molecule, and Δ*r* is the bin size.

In our earlier work (Karczyńska et al., 2018), *P* ^*calc*^ was a sum of Gaussians, each centered on a given C^*α*^⋯ C^*α*^ distance, with a fixed standard deviation. As a result, the calculated *P*(*r*) curves were slightly shifted to the right and the parts corresponding to the small distances were too small. Moreover, the solvation shell was not taken into account. Later, we revised the formula to replace the Gaussians with log-normal functions and to introduce an estimate of solvation shell, as given by Eq. 16.

$$P^{\mathrm{calc}}\left(r_{k}\right)=\frac{1}{A}\underset{i}{\sum }\underset{j<i}{\sum }\exp\left[-\frac{{\left(\ln r_{ij}-\ln r_{k}\right)}^{2}}{2\sigma_{ij}^{2}}\right]$$ (16)

$$A=\Delta r\overset{M}{\underset{k=1}{\sum }}\underset{i}{\sum }\underset{j<i}{\sum }\exp\left[-\frac{{\left(\ln r_{ij}-\ln r_{k}\right)}^{2}}{2\sigma_{ij}^{2}}\right]$$ (17)

$$\sigma_{ij}=\frac{{r{}^{\circ}}_{i}+{r{}^{\circ}}_{j}}{r_{ij}}$$ (18)

$${r{}^{\circ}}_{i}=\frac{\rho_{i}}{s}+\sigma_{max}+\frac{\left(\sigma_{min}-\sigma_{max}\right)\left(x_{i}+1\right)}{2}$$ (19)

where *r*_*ij*_ is the distance between the C^*α*^ atoms of residues *i* and *j* in the calculated conformation, *σ*_*ij*_ is the standard deviation of the respective Gaussian, *ρ*_*i*_ is the Stokes’ radius of residue *i*, *σ* *_min_* and (*σ* *_min_* +*σ* *_max_*)/2 are minimum and maximum size of the solvation shell of residue *i* and *x*_*i*_ is equal to 0 if residue *i* has no neighbors and 1 if its solvation shell is maximally filled with neighboring residues. *A* is the factor normalizing the calculated probability to 1.

The above implementation of SAXS restraints to run data-assisted simulations seems to be the only one for a coarse-grained model. Grudinin et al. (2021) have recently reported an implementation of their Pepsi-SAXS/SANS method to run SAXS-data-assisted simulations; however, they use all-atom representation.

### 2.4 UNRES web server

The UNRES web server (Czaplewski et al., 2018) is available at https://unres-server.chem.ug.edu.pl. No registration is required to run jobs; however, registered users have access to past jobs. The peptide-protein and protein-protein docking functionality (UNRES-Dock) has recently been added to the server (Krupa et al., 2021). The following types of jobs can be run with the server:

1. Energy minimization of the input structure.
2. Molecular dynamics (MD) simulations. These can be run in both canonical (NVT) and microcanonical (NVE) mode. The Berendsen et al. (1984) and the Langevin thermostats are available to run NVT simulations. A trajectory movie is displayed after the job is completed and fluctuations of C^*α*^ positions are calculated and visualized. If a reference structure has been input, the variation of root mean square deviation (RMSD) from the reference structure with time is calculated and displayed.
3. Replica exchange (REMD) and multiplexed replica-exchange molecular dynamics (MREMD) simulations. Runs of this type are aimed at the modeling of the conformational ensembles, in particular the representative conformations at the selected temperatures. A protocol that consists of a production (M)REMD run, processing the results with WHAM (Kumar et al., 1992), and cluster analysis (Murtagh and Heck, 1987) developed in our earlier work (Krupa et al., 2016) is applied. The final representative structures are converted to all-atom structures by using PULCHRA (Rotkiewicz and Skolnick, 2008) and SCWRL (Wang et al., 2008).
4. Docking-type runs. These runs are aimed at predicting the structures of peptide-protein or protein-protein complexes and are always carried out in the (M)REMD mode. Details of the UNRES docking protocol are described in (Krupa et al., 2021).

The user can select the force field. The input structure can be read from a Protein Data Bank (Berman et al., 2000) (PDB) file (in this case the sequence is not input separately but is taken from the PDB file) or a starting extended or randomly-generated structure can be specified. The present version of the server does not repair incomplete PDB structures; for this purpose, MODELLER (Fiser and Šali, 2003) or other software has to be used. Secondary-structure restraints can also be input. The input can be specified in a simpler way by using the “Basic” options or in a more advanced way by using the “Advanced” options. Details of specifying the input are in the “Input data,” “Tutorial,” and “UNRES-Dock tutorial” sections of the server. Apart from the visual output, the users (both unregistered and registered) can download all the output files (main output file, run summary files, PDB files, etc.) produced by the server.

In the first version of the UNRES web server (Czaplewski et al., 2018) only SAXS-assisted simulations were enabled, while NMR- and XL-MS-data assisted simulations have been enabled in the present version. These features are described in Section 3.1; for consistency, we have also included a short description of the SAXS-data assisted feature in that section. The reader is referred to our earlier work (Czaplewski et al., 2018) for the description of the other functions of the UNRES server.

## 3 Results

### 3.1 Implementation of the new features in the UNRES server

The new scale-consistent NEWCT9P force field (Liwo et al., 2019) has been included in the upgraded UNRES server. The old OPT-WTFSA-2 force field (Krupa et al., 2017a) can still be used. It should be noted that the new optimized code runs only the NEWCT-9P force field and selecting OPT-WTFSA-2 means running the slower code.

SAXS restraints in the form of *P*(*r*) as described in Section 2.3.3 are read from the appropriate ASCII text file supplied by the user. The data format is that provided by the CRYSOL program (Svergun et al., 1995).

NMR restraints described in Section 2.3.1 can be read in the plain-text format, in which the restraints for the NMR-assisted targets were provided during the CASP experiments (Sala et al., 2019), in the NMR-star format (Ulrich et al., 2019), in which most of the NMR data are deposited in the PDB, or in the NMR Exchange Format (NEF) (Gutmanas et al., 2015), which has become the standard. If both angular and distance restraints are present in a NMR-data file the user can choose to use only the distance restraints. Details and examples of NMR-restraint input are included in the “Input data” section of the UNRES web server page.

Crosslink restraints (described in Section 2.3.2) are supplied by the user in ASCII files. The crosslink-restraint type (see section Section 2.3.2) is selected from the menu. Details are described in the “Input data” section of the UNRES web server page.

### 3.2 Examples

#### 3.2.1 NMR examples

##### 3.2.1.1 Unambiguous NMR distance and angular restraints

**FIGURE 3:**
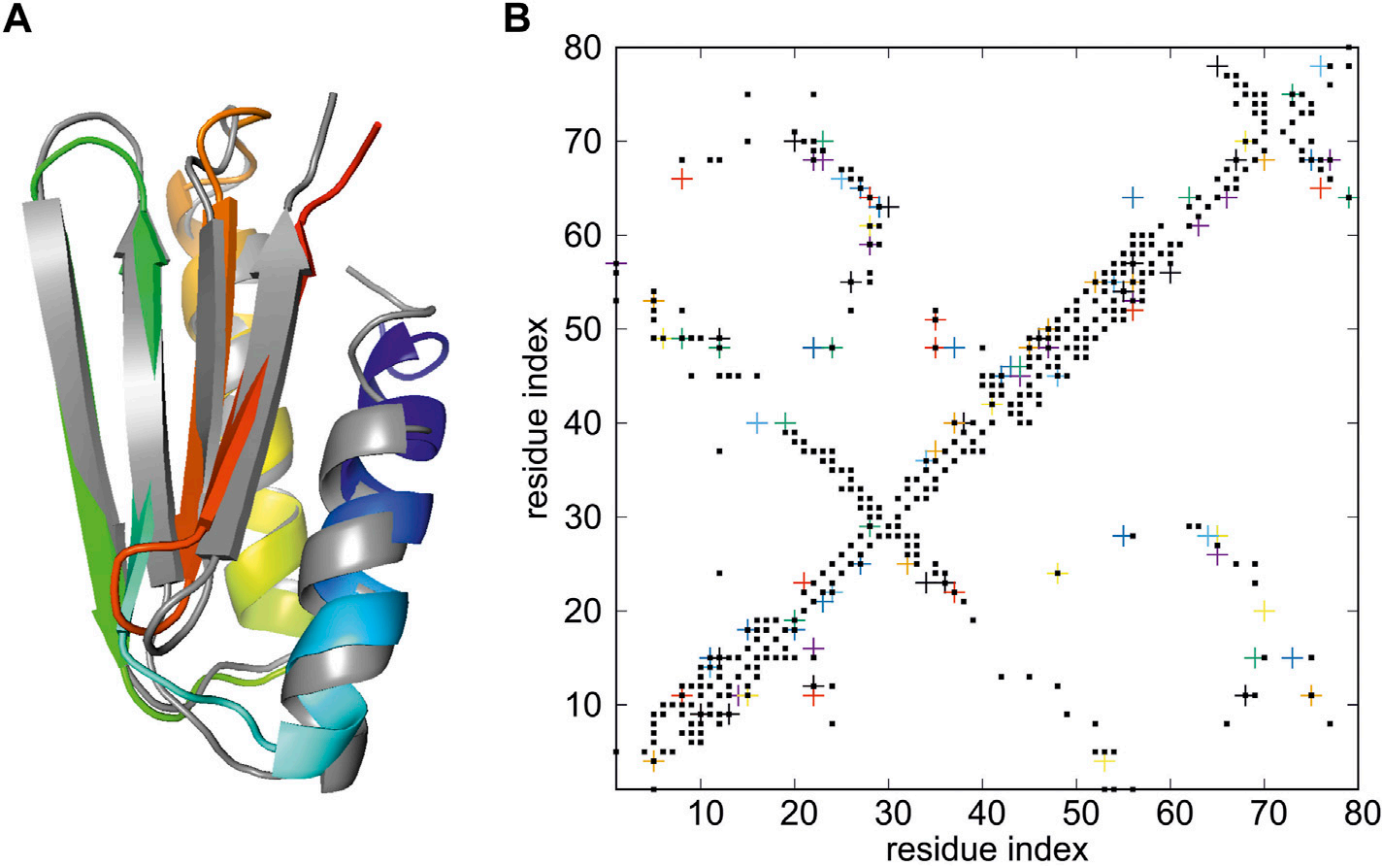
**(A)** Superposition of the model 1 of the structure of *de novo* designed Foldit3 protein (PDB: 6msp) simulated with NMR-data-assisted UNRES, using the expert-edited restraints deposited at the 6msp PDB entry (gray) and the experimental structure (colored from blue to red from the N- to the C-terminus). The RMSD and GDT_TS values are 2.03 Å and GDT_TS of 76.88, respectively. **(B)** The proton-proton contacts in the calculated structures (black squares) superposed on those found from NMR experiments (colored crosses).

We used the restraint data of the *de novo* designed Foldit3 protein (Koepnick et al., 2019) (PDB: 6msp), with distance restraints taken from the respective PDB entry (NMR restraints v2). The unstructured 17-residue N-terminal tag has been removed and the restraints have been edited accordingly. A total of 1,279 restraints were included in the calculations. The target is an 81-residue *α*+*β* protein and was one of the test-set protein used to test the NMR-data-assisted implementation of UNRES (Lubecka and Liwo, 2022). The server example data specify an 8-trajectory REMD run with replica temperatures of 250, 260, 270, 280, 290, 300, 315, and 330 K, respectively. Each trajectory consisted of Langevin-dynamics 2,000,000 steps with a 9.78 fs step length and was started from a randomly-generated conformation. This is a much less resource-demanding run compared to the 144-replica 20,000,000-step Hamiltonian Replica Exchange (HREMD) run for this protein performed to test NMR-data-assisted UNRES (Lubecka and Liwo, 2022). The conformation of the first cluster that fits the NMR data best (model 1) has C^*α*^ RMSD from the experimental structure of 2.03 Å and Global Distance Test Total Score GDT_TS (Zemla, 2003; Moult et al., 2013) of 76.88, compared to 1.61 Å and 87.19, respectively in (Lubecka and Liwo, 2022). The superposition of the calculated on the experimental structure is shown in Figure 3A, while the proton-proton contact map corresponding to the model superposed on that resulting from the NMR measurements is shown in Figure 3B.

##### 3.2.1.2 Ambiguous NMR distance restraints

**FIGURE 4:**
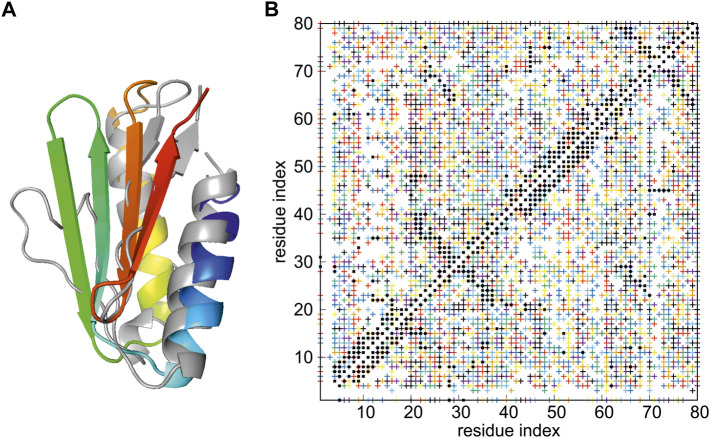
**(A)** Superposition of the best model of the structure of *de novo* designed Foldit3 protein simulated with NMR-data-assisted UNRES, using highly ambiguous distance restraints (gray), and the experimental 6msp structure (colored from blue to red from the N- to the C-terminus). The RMSD and GTD_TS values are 3.87 Å of 64.06, respectively. **(B)** The proton-proton contacts in the calculated structures (black squares) superposed on those found from NMR experiments (colored crosses, each color corresponding to a group of ambiguous restraints).

As in Section 3.2.1.1, the protein is the *de novo* designed protein Foldit3 (Koepnick et al., 2019) (PDB: 6msp), which was the data-assisted CASP13 target n1008. The restraint set, containing a total of 26,626 restraints, was provided to the CASP13 participants at https://predictioncenter.org/download_area/CASP13/extra_experiments by G.T. Montelione. These are raw data prior to expert editing. The number of possible assignments per peak exceeds 100 for some of the peaks; moreover, more than a half of them are violated by the experimental 6msp structure (Lubecka and Liwo, 2022). These data were used to test the NMR-data-assisted functionality of the UNRES package in our previous work (Lubecka and Liwo, 2022). With the server version, 12 REMD trajectories were run at 260, 262, 266, 271, 276, 282, 288, 296, 304, 315, 333, and 370 K, respectively. These temperatures have been determined by using the Hansmann algorithm (Trebst et al., 2006), which maximizes the number of walks in the temperature space. Each trajectory consisted of 2,000,000 steps with a 9.78 fs length and was started from a randomly-generated conformation. The representative conformation of the best model has C^*α*^ RMSD of 3.87 Å and GDT_TS of 64.06, compared to 1.66 Å and of 80.00 for the full-blown run with 144 HREMD trajectories, each consisting of 20,000,000 steps (Lubecka and Liwo, 2022). The calculated structure is superposed on the experimental structure in Figure 4A, while the proton-proton contact map corresponding to the model superposed on that resulting from the NMR measurements is shown in Figure 4B. It should be noted that a structure reasonably close to the experimental structure was obtained despite the very high ambiguity of the NMR restraints (Figure 4B) and limited computational resources applied.

### 3.3 XL-MS example

The example is horse heart cytochrome (PDB: 1hrc), a 105-residue *α*-protein, which was one of the proteins used in our previous work (Kogut et al., 2021) to test the XL-MS-data-assisted functionality of the UNRES package. As opposed to that work, no secondary-structure restraints were imposed in the calculations with the UNRES server. The data from lysine-lysine crosslinking experiments were taken from the Xlink Analyzer database (Kosinski et al., 2015), the original source being described in (Seebacher et al., 2006). There are a total of 24 crosslinks. The run consisted of 12 REMD trajectores, 2,000,000 steps per trajectory. The temperatures were set as in Section 3.2.1.2. Each trajectory was started from a randomly-generated conformation. Even with the limited server resources, the GDT_TS is 26.68, compared to 34.90 with the 48 MREMD trajectories each consisting of 20,000,000 steps and secondary-structure restraint imposed (Kogut et al., 2021).

#### 3.3.1 Timing

The calculations of the first NMR example (unambiguous NMR data), take 1.2 ms/MD step with two cores/trajectory of an 20-core Intel(R) Xeon(R) CPU E5-2640 v4 @ 2.40 GHz processor when using the optimized code, both running the scale-consistent NEWCT-9P force field. With the old code, the calculations take 3.5 ms/MD step. Thus, code optimization resulted in an about 3-fold reduction of execution time. With the same settings, the calculations of the second NMR example (ambiguous NMR data of the same protein) take 1.7 ms/MD steps when using the optimized and 8.7 ms/MD step with the non-optimized code, respectively. Thus, with the optimized code, the execution time increases only by about 40%, while it increases more than twofold for the non-optimized code. The optimized code is over five times faster than the non-optimized code. The increase of the execution time with optimized code is due to a great total number of ambiguous distance restraints (26,626). With the old code the exponential terms in Eq. 9 corresponding to all these distances were evaluated, while only those that make near-zero contributions to the penalty function are not computed.

For the crosslink example, the calculations took 0.7 ms/MD step with four cores/trajectory of an 20-core Intel(R) Xeon(R) CPU E5-2640 v4 @ 2.40 GHz processor with the optimized code and 2.3 ms/MD step with the old code, giving an over 3-fold speed-up. It should be noted that the examples analyzed here are small proteins, for which full-blown data-assisted calculations are doable with the UNRES web server.

## 4 Conclusion and outlook

We upgraded the UNRES server to include the new scale-consistent variant of the UNRES force field (Liwo et al., 2019) that, owing to the introduction of the dependence of the backbone-virtual-bond torsional and correlation potentials on backbone-virtual-bond angles handles the *β*-strand and loop geometry and, consequently, that of the *β*- and *α*+*β*-proteins better than the old version of UNRES. We have also replaced the old code with the recently optimized code (Sieradzan et al., 2022a), this speeding up the calculations at least twice.

The existing SAXS-data-assisted functionality has been upgraded by replacing the old penalty function with one that reproduces the asymmetry of the distance distribution and takes into account the solvation shell in a simple empirical manner (Eqs 15, 16). Two new functionalities recently introduced to the UNRES package were added to the server version, namely NMR- and XL-MS-data-assisted simulations. The NMR penalty function is based on our recently developed ESCASA algorithm (Lubecka and Liwo, 2019) to estimate proton positions from coarse-grained geometry analytically. Highly ambiguous restrains can be handled (Lubecka and Liwo, 2022). The XL-MS restraints include our recently developed pseudopotentials that restrain the distances in a stricter manner than plain distance boundaries or the C^*α*^-distance based statistical potentials (Kogut et al., 2021).

The introduced modifications have extended the scope of simulations possible to run with the UNRES server. In particular, its capacity to handle contradictory NMR and XL-MS restraints and ambiguous NMR restraints enables the user to run data-assisted simulations of intrinsically-disordered proteins, in which case the restraints can happen to be ambiguous and often do not pertain to a single structure (Bonomi et al., 2016). To our knowledge, the UNRES web server is the only publicly available server for protein simulations that enables the users to run full-blown data-assisted simulations that include the restraints from NMR, XL-MS and SAXS experimental data.

The examples presented in Section 3.2 demonstrate that reasonable results can be obtained, with limited resources, using state-of-the art UNRES and conformational-search methods. However, work on improvement of the UNRES force field and the search method, in particular on the ensemble-oriented conformational search, is underway in our laboratory. These modifications will be gradually introduced to the server version.

## Data Availability

The raw data supporting the conclusion of this article will be made available by the authors, without undue reservation.
